# Activation of the *Arabidopsis thaliana* GCN2-eIF2α module by salicylic acid: Biochemical and phenotypic similarities between *gcn2* mutant and wild type

**DOI:** 10.17912/micropub.biology.001771

**Published:** 2025-09-26

**Authors:** Morgan Wynn, Ansul Lokdarshi, Daniel Rincon Diaz

**Affiliations:** 1 Department of Biology, Valdosta State University, Valdosta, Georgia, United States

## Abstract

Plants rely on arsenal of defenses to combat numerous stresses. Salicylic acid (SA) is a crucial hormone in plant defense, yet its role in translational regulation is underexplored. We investigated the SA signaling through the stress sentinel protein kinase, GCN2 and its substrate, eIF2α, in *Arabidopsis thaliana* . We show that the GCN2 dependent eIF2α phosphorylation in response to SA is regulated by the photosynthetic state of the plant. Notably, *GCN2* knockout mutant exhibits wild-type like growth under SA stress, suggesting limited role of the GCN2-eIF2α module in the management of physiological defects under SA stress.

**Figure 1. Effect of SA stress on GCN2 mediated eIF2α phosphorylation and growth physiology:**
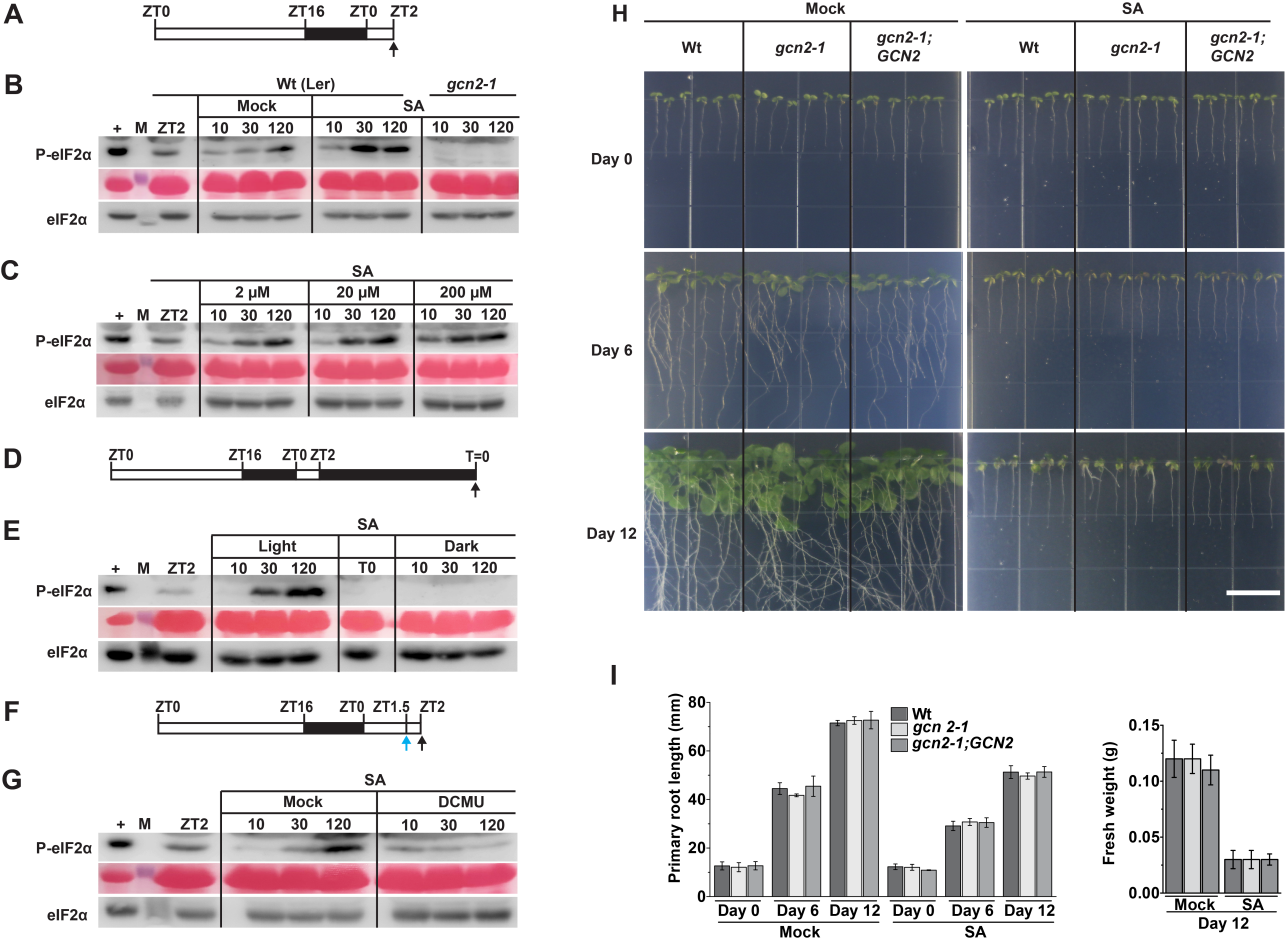
**(A)** Schematic of the 16 h light: 8 h dark growth regime of Arabidopsis seedlings and time of treatments (black arrow) starting at ZT2 (zeitgeber time) for panel B. **(B)** Top -Immunoblot showing eIF2α phosphorylation (P-eIF2α) in wild-type Landsberg (Wt) and *gcn2-1* mutant seedlings at pre- (ZT2) and post-treatment with either 0.001% DMSO (Mock) or 20 µM Salicylic acid (SA) at 10, 30 and 120 minutes. Middle: Ponceau stained blot with Rubisco large subunit representing equal loading of total protein. Bottom: Immunoblot showing total eIF2α. (+) indicates a positive control for P-eIF2α and (M) denotes a molecular weight marker for P-eIF2α/eIF2α (~38kDa). **(C)** P-eIF2α/ eIF2α in Wt seedlings treated 2, 20 and 200 µM SA. **(D)** Schematic of growth regime showing 24 hour dark acclimation starting at ZT2 and time of sampling before SA treatment under dark (T0) for panel (E). **(E)** P-eIF2α/ eIF2α in Wt seedlings treated 20 µM SA under light or dark. **(F)** Schematic of growth regime showing time of pretreatment (ZT1.5/blue arrow) with either DMSO (Mock) or DCMU thirty minutes prior to 20 µM SA treatment (black arrow) at ZT2 for panel (G). **(G)** P-eIF2α/ eIF2α in Wt seedlings after treatments as described in (F). **(H)** Wt, *gcn2-1* , and *gcn2-1; GCN2* seedlings at day 3, day 6 and at day 12 on growth medium supplemented with either DMSO (Mock) or 20 µM SA. Scale bar 2 cm. **(I)** Primary root length (left) and Fresh weight (right) of Wt, *gcn2-1* , and *gcn2-1; GCN2* seedlings from panel (H). Error bars represent standard deviation from four biological replicates with n>30 seedlings per biological replicate.

## Description

Plant cells possess a multitude of regulatory programs to adjust gene expression and physiology in response to diverse environmental challenges. Among the key stress management programs, regulation at the level of protein synthesis (translational control) has gathered considerable interest in recent years (Hershey et al., 2019; Wu et al., 2024). Translational control comprises the biochemical and cellular mechanisms that modulate protein synthesis efficiency by adjusting the initiation, elongation or termination of ribosomes as they translate mRNAs into proteins (Hershey et al., 2019; Urquidi Camacho et al., 2020). The translation initiation factor eIF2 plays a central role in translational control because it is part of a larger protein complex that delivers tRNA-Met to initiating mRNAs (Hinnebusch, 2014). Phosphorylation of the alpha (α)-subunit of eIF2 by the highly conserved protein kinase, General Control Nonderepressible 2 ( GCN2 ) represents a key regulatory node for controlling translational initiation across all eukaryotes (Kimball, 1999; Wek, 2018). Phosphorylation of eIF2α (hereafter referred to as P-eIF2α) generally leads to a reduction in global translation, helping the cell conserve energy during the stress period. In *Arabidopsis thaliana* , GCN2 ( AT3G59410 ) has been shown to induce P-eIF2α ( AT2G40290 / AT5G05470 ) in response to a variety of abiotic (e.g., high light, cold salt, hypoxia, nutrient), biotic (e.g., *Botrytis cinerea* ) and xenobiotic (e.g., herbicides, H _2_ O _2_ ) stresses (Berrocal-Lobo et al., 2020; Cho et al., 2022; Cui et al., 2025; Lageix et al., 2008; Lokdarshi et al., 2020a; Lokdarshi et al., 2020b; Lokdarshi & von Arnim, 2022; Lokdarshi et al., 2022; Zhang et al., 2008).

Conventionally, GCN2 activation in metazoans and yeast relies on binding to uncharged tRNAs under amino acid starvation conditions (Dever & Hinnebusch, 2005; Dong et al., 2000); however, this hypothesis remains to be thoroughly tested in plants under various stress conditions. In a previous report, Lokdarshi et al., 2020a, showed that Arabidopsis GCN2 is activated by reactive oxygen species (ROS) emanating from chloroplasts under numerous stresses such as excess light, herbicides, cold and salt (Lokdarshi et al., 2020a; Lokdarshi et al., 2020b). More recently, Cui et al., 2025 extended this model by showing that ROS and photosynthetic state of the plant mediate GCN2 activation under macronutrient starvation (Cui et al., 2025). Taken together, these studies suggest that in plants, GCN2 activation may not be exclusively dependent on uncharged tRNA sensing and binding. Instead, both uncharged tRNAs and photosynthetically generated ROS may serve as upstream signals in a context-dependent or potentially integrated manner, indicating a boarder and more versatile stress-sensing role for the plant GCN2-eIF2α pathway. In the present study, we aimed to test whether regulation of the GCN2-eIF2α pathway by the photosynthetic state of the plant is conserved under salicylic acid (SA) stress, and to assess the impact of the *GCN2* loss-of-function on plant health during prolonged SA treatment.

SA is a notable defense hormone involved in plant immune responses and systemic acquired resistance (Spoel & Dong, 2024). A prior study in Arabidopsis showed that SA induces P-eIF2α at 4 hours and 12 hours in the wild-type Landsberg seedlings (Lageix et al., 2008). Here, we show that SA triggers P-eIF2α rapidly within 30 – 120 minutes in a GCN2 dependent manner (Fig 1. A-B). The lack of P-eIF2α signal in the *gcn2* mutants aligns with previous findings involving other stresses as well, further reinforcing GCN2 's essential role as the “stress sentinel kinase” in plants. Having established that SA rapidly activates GCN2 and triggers P-eIF2α, we hypothesized that modulating SA concentrations would result in a corresponding change in P-eIF2α signal. This dose-dependent GCN2 activation has been previously documented under various stress conditions (e.g. H _2_ O _2_ , chlorosulfuron, NaCl) (Lokdarshi et al., 2020a; Lokdarshi et al., 2020b). However, in our study, 200 µM SA-treated seedlings did not exhibit a significantly greater P-eIF2α signal compared with the 2 or 20 µM treatment group at 30 and 120 minutes (Fig 1. C). This lack of linear response may reflect the existence of an optimal SA concentration range required for efficient GCN2- eIF2α mediated signaling in Arabidopsis seedlings.

The GCN2 activation has been shown to be light-dependent under numerous stress conditions (Lokdarshi et al., 2020a; Lokdarshi et al., 2020b; Lokdarshi et al., 2022). This observation is believed to be caused by the over accumulation of reactive oxygen species (ROS), mainly produced in the chloroplast during photosynthesis (Lokdarshi et al., 2020a; Lokdarshi et al., 2020b). As a result, in the absence of light, we would not expect to observe P-eIF2α. We discovered that dark acclimated seedlings showed no detectable P-eIF2α in contrast to those treated with SA under light, which displayed robust P-eIF2α induction (Fig 1. D-E), suggesting that GCN2 activity under SA stress is dependent on the photosynthetic state of the plant. We subsequently checked the involvement of photosynthetic ROS in the activation of GCN2 by using a photosynthetic inhibitor, DCMU (3-(3,4-dichlorophenyl)-1,1-dimethylurea). DCMU blocks electron transfer from photosystem II to plastoquinone, thereby lowering the accumulation of chloroplastic ROS (Kruk & Karpinski, 2006; Lokdarshi et al., 2020a; Mateo et al., 2004). We found that seedlings pretreated with DCMU exhibit reduced P-eIF2α compared to mock-treated control at 120 minutes (Fig 1. F-G). This finding, together with our results indicating light dependence, suggests that photosynthesis plays a key role in GCN2 activation under SA-induced stress.

Lastly, we investigated how the biochemical responses to SA stress manifest in phenotypic changes in Arabidopsis seedlings. Subsequently, we tested the hypothesis that GCN2 function is critical for the survival responses towards SA stress by assessing the phenotypic differences between wild-type and *gcn2* mutant seedlings. Surprisingly, we found similar phenotypic behavior between the wild-type, *gcn2-1* mutant, and a complementation line ( *gcn2-1; GCN2* ) in a primary root length and fresh weight assay across all treatments and time points (Fig 1. H-I). These results suggest that GCN2 may not be the sole or even primary signaling component involved in mediating growth responses under prolonged SA stress. Future investigations integrating alternative kinase pathways and translational repression will be essential to fully understand the phenotypic behavior of *gcn2* mutants during prolonged SA exposure.

## Methods

***Plant material, growth condition and stress treatments***

Seeds of *Arabidopsis thaliana* ecotype Landsberg erecta (Ler-0) and homozygous *gcn2-1* mutant in Ler-0 (Genetrap line GT8359) (Lageix et al., 2008; Zhang et al., 2008) and *gcn2-1; GCN2* complementation with *GCN2* under native promoter (Lageix et al., 2008) were sterilized and stratified in dark at 4°C for 48 hours. Seeds were spotted on a ½-strength Murashige-Skoog (MS) (MilliporeSigma, Cat# M5524) containing 0.65% Phytoagar (Bioworld, Cat# 40100072-2). Germination and growth were under the standard long-day cycle of 16 hours light (80 ± 10μEin m−2 s−1) and 8 hours dark at 22°C with 50% humidity. For SA treatment, 12-day-old seedlings grown on horizontal plates (roots inside the medium) were treated with either Dimethyl Sulfoxide (DMSO -VWR, Cat# 0231-500ML) as mock or SA (ThermoFisher, Cat# A12253) in 0.001% (v/v) DMSO at the desired time e.g., Zeitgeber time 2 (ZT2). Seedlings receiving no stress treatment were collected at ZT2 before the treatments. Whole seedlings were collected, and flash frozen in liquid N2. For sampling with a photosynthetic inhibitor, seedlings were pretreated with 3-(3,4-dichlorophenyl)-1,1-dimethylurea (DCMU) (MilliporeSigma, Cat# D2425) thirty minutes prior to ZT2 and the subsequent application of SA or mock treatment. For SA treatment in dark, seedling plates were shifted to dark for 24 hours starting at ZT2. Treatment and sampling were done under low levels of safe green light.

***Protein extraction and Immunoblot analysis***

Protein extraction and P-eIF2α/ eIF2α immunoblotting experiments were performed as described previously by Lokdarshi et al. (2020a) with minor modifications. Total protein extraction was performed by grinding seedlings using bead mill homogenizer 24 (Fisher Scientific, Cat # 15340163) with 80-120 μl of freshly prepared lysis buffer containing 1 M urea (IBI Scientific, Cat# IB72064), 25 mM Tris-HCl (pH 7.5), 75 mM NaCl (J.T. Baker, Cat# 4058-01), 5% (v/v) glycerol (Fisher Scientific, Cat# G33-500), 0.5 mM EGTA (MilliporeSigma, Cat# E3889-25G), 0.5 mM EDTA (Fisher Scientific, Cat# S311-100), 2% (w/v) polyvinylpyrrolidone (MilliporeSigma, Cat# PVP-40), and 2 mM β-mercaptoethanol (MilliporeSigma, Cat# M-6250, Lot# 58H0066), supplemented with 1X Protease and Phosphatase inhibitor cocktail (ThermoFisher; Cat# PIA32959). Total protein was quantified using Bradford Plus Protein Assay Kit as per manufacturer's instructions (ThermoFisher, Cat # 23236).

50 µg total protein was separated on a 12% (w/v) SDS-Polyacrylamide gel. After the electrophoretic run, the gel was incubated in 1X transfer buffer (20% (v/v) methanol, 25 mM tris base, 190 mM glycine, 0.01% (w/v) SDS) for 30 minutes at room temperature prior to initiating the electroblotting. Proteins were electroblotted onto a 0.45 µm polyvinylidene difluoride (PDVF) membrane (MilliporeSigma, Cat# IPVH00010) for 16 hours at 4°C. Blocking of the membrane was performed for 1 hour with 1X TBST buffer (20 mM Tris buffer saline (pH 7.6), 0.07% Tween-20) containing 10% non-fat dry milk (RPI, Cat# M17200) and 0.2% (w/v) bovine serum albumin (BSA) (Fisher Scientific, Cat# BP9706-100) at room temperature. Membrane was washed with 1X TBST for 10 minutes (8 repeats) and incubated overnight with a monoclonal anti-eIF2S1 (phospho-S51) antibody (Abcam, cat# 32157) diluted to 1:2500 in 1 X TBST with 5% (w/v) BSA. Following washing, the membrane was incubated with horseradish peroxidase-conjugated anti-rabbit IgG (ThermoFisher, Cat# 32260) diluted to 1:5000 in 1 X TBST containing 1/10 dilution of the blocking buffer for 1 hour at room temperature. After washing with 1X TBST for 10 minutes (8 repeats), chemiluminescence was performed as per manufacturer's protocol (WesternBright Quantum HRP substrate, advansta, Cat# K-12042-D20) and blots were imaged using a GE ImageQuant LAS-4000 Multi-Mode Imager. For total eIF2α, immunoblot experiments were performed with a rabbit polyclonal eIF2α antibody (a gift from Dr. Karen Browning, University of Texas, Austin) as described previously in Lokdarshi et al. 2020a.

***Primary root length, Fresh weight measurements, and Statistical analysis***

For phenotype characterization, 3-day-old vertically grown seedlings on ½ strength MS media were transferred to either a medium containing 20 μM SA or 0.001% DMSO as mock. Photographs were taken on day 0 (the day of the transfer), day 6 and day 12. Primary root length was measured with the Image J program (ver. 1.4). Seedlings were collected after imaging on day 12 for fresh weight measurements. All statistical analyses for test of significance were performed using GraphPad Prism version 5.

## Data Availability

Description: P-eIF2α Immunoblot Signal Quantification. Resource Type: Dataset. DOI: https://doi.org/10.22002/w2kjv-j0223
